# Population Genetics of *GYPB* and Association Study between GYPB*S/s Polymorphism and Susceptibility to *P. falciparum* Infection in the Brazilian Amazon

**DOI:** 10.1371/journal.pone.0016123

**Published:** 2011-01-24

**Authors:** Eduardo Tarazona-Santos, Lilian Castilho, Daphne R. T. Amaral, Daiane C. Costa, Natália G. Furlani, Luciana W. Zuccherato, Moara Machado, Marion E. Reid, Mariano G. Zalis, Andréa R. Rossit, Sidney E. B. Santos, Ricardo L. Machado, Sara Lustigman

**Affiliations:** 1 Departamento de Biologia Geral, Universidade Federal de Minas Gerais, Belo Horizonte, Brazil; 2 Laboratório de Pesquisa em Hemoterapia- Hemocentro Campinas, São Paulo, Brazil; 3 Centro de Investigação de Microrganismos, Faculdade de Medicina de São José do Rio Preto, São José do Rio Preto, Brazil; 4 Laboratory of Immunochemistry, Lindsley F. Kimball Research Institute, New York Blood Center, New York, New York, United States of America; 5 Laboratório de Infectologia e Parasitologia Molecular, Universidade Federal do Rio de Janeiro, Rio de Janeiro, Brazil; 6 Laboratório de Genética Humana e Médica, Universidade Federal do Pará, Belem, Brazil; 7 Laboratory of Molecular Parasitology, Lindsley F. Kimball Research Institute, New York Blood Center, New York, New York, United States of America; Université Pierre et Marie Curie, France

## Abstract

**Background:**

Merozoites of *Plasmodium falciparum* invade through several pathways using different RBC receptors. Field isolates appear to use a greater variability of these receptors than laboratory isolates. Brazilian field isolates were shown to mostly utilize glycophorin A-independent invasion pathways via glycophorin B (GPB) and/or other receptors. The Brazilian population exhibits extensive polymorphism in blood group antigens, however, no studies have been done to relate the prevalence of the antigens that function as receptors for *P. falciparum* and the ability of the parasite to invade. Our study aimed to establish whether variation in the GYPB*S/s alleles influences susceptibility to infection with *P. falciparum* in the admixed population of Brazil.

**Methods:**

Two groups of Brazilian Amazonians from Porto Velho were studied: *P. falciparum* infected individuals (cases); and uninfected individuals who were born and/or have lived in the same endemic region for over ten years, were exposed to infection but have not had malaria over the study period (controls). The GPB Ss phenotype and GYPB*S/s alleles were determined by standard methods. Sixty two Ancestry Informative Markers were genotyped on each individual to estimate admixture and control its potential effect on the association between frequency of GYPB*S and malaria infection.

**Results:**

GYPB*S is associated with host susceptibility to infection with *P. falciparum*; GYPB*S/GYPB*S and GYPB*S/GYPB*s were significantly more prevalent in the in the *P. falciparum* infected individuals than in the controls (69.87% vs. 49.75%; *P*<0.02). Moreover, population genetics tests applied on the *GYPB* exon sequencing data suggest that natural selection shaped the observed pattern of nucleotide diversity.

**Conclusion:**

Epidemiological and evolutionary approaches suggest an important role for the GPB receptor in RBC invasion by *P. falciparum* in Brazilian Amazons. Moreover, an increased susceptibility to infection by this parasite is associated with the GPB S+ variant in this population.

## Introduction

Specificity of invasion *of Plasmodium falciparum* merozoites into human red blood cells (RBCs) was the first indication that malaria parasites possess ligands that recognize and interact exclusively with receptors on the surface of the host RBCs [1]. The specificity of malaria parasites for RBC depends on a number of ligand-receptor interactions that are dynamic in *P. falciparum* and provide a greater flexibility to the parasite to overcome the variability in host RBCs and to evade immune responses. The *P. falciparum* merozoites invade through several pathways using different RBC receptors. Receptors include the glycophorin A, B and C (GPA, GPB and GPC), Band 3 and others (Receptors Y, E, Z and X), whose molecular identity has not yet been determined [2]. Field isolates have shown even greater variability than the laboratory strains in which the known invasion pathways have been defined [3]–[9]. Our initial studies of field isolates from Mato Grosso, Brazil have revealed that these parasites differ from common laboratory strains, and mostly utilized GPA-independent invasion pathways – the so-called alternative invasion pathways via receptors such as GPB, Receptor Y, Receptor Z and/or others [2].

The genetic polymorphisms of glycophorins or other known receptors play an important role in the resistance to the invasion of erythrocytes by *P. falciparum* [10]–[19]. In hyperendemic malaria regions of Papua New Guinea deletions in Band 3 (*SLC4A1*Δ*27*) or in GPC (Ge phenotype, *GYPCΔ*ex3) are highly prevalent and these RBC phenotypes confer selective advantage on morbidity [13]–[19]. *In vitro* invasion of RBCs that have GPC deficiency is significantly reduced but not eliminated [10], [11], [20]–[23].

The polymorphisms identified so far in the two other known *P. falciparum* glycophorin receptors, GPA (GenBank M60707) and GPB (GenBank M60708), have been shown to be only mildly associated with the efficiency of *P. falciparum* invasion and they conferred only partial protection against invasion of RBCs [24], [25]. For example, the invasion of *P. falciparum* malaria of En(a−) (lack of GPA) and S−s−U− (lack of GPB) RBCs is significantly reduced but not eliminated [10], [11], [20]–[23], presumably because the parasites can use one or more of the other RBC receptors for invasion.

The *GYPA* and *GYPB* genes code for Type I membrane RBC proteins that carry antigens of the MNS blood group system. *GYPA* has two codominant allelic forms, which determine the M or N antigens at the N-terminus of GPA (^1^SSTTG^5^ for M and ^1^LSTTE^5^ for N). GPB is identical to GPA^N^ for the first 26 amino acids and, thus, also encodes the N antigen at the N-terminus. *GYPB* also has two codominant alleles: GYPB*S and GYPB*s corresponding to S and s antigens, respectively, on the RBC surface. The Ss antigens are defined by an amino acid change at position 29 [Met(S)/Thr(s)] of GPB and are displayed as the S+s−; S−s+ or S+s+ phenotypes [26]. In addition to their sequence homology, *GYPA* and *GYPB* recombination and gene conversion hotspots have been identified, generating on the RBC surface many different hybrid *GYP* gene products that encode GPB bearing low-prevalence antigens like He and Dantu [27]. The GP.Dantu glycophorin is specified by a hybrid gene whose N-terminal sequence is encoded by the *GYPB* gene and C-terminal sequence by the *GYPA* gene. In this hybrid, the genomic breakpoint is located in its intron 4, a composite of *GYPB* and *GYPA* [28]. The hallmark of GPB.He antigen is the change of the N-terminal sequence (from ^1^LSTTE^5^ for N to ^1^WSTSG^5^ for He) that abolishes the expression of the common N antigen. GP.He isoform of GPB may or may not carry the S or the s antigen depending on whether it associates with mutations that affect the splicing of exon 4 [29]. Dantu invariably expresses s, albeit weakly. The Dantu variant is prevalent in Africans; 4% vs. 0% in Europeans [30], [31]. The *in vitro* invasion of GP.Dantu RBCs by *P. falciparum* is severely compromised [30], [32].

Some other *GYPB* nucleotide changes are known to influence the expression of S or s antigens on the RBCs [27], [33]. The GPB U antigen is defined by aa 33–39 [34]. Thus, a deletion of *GYPB* exons 2–5 results in S−s−U− phenotype; absence of GPB on the RBCs. Although, the GPB U− RBCs are also S−s−, approximately 16% of S−s− RBCs are U+ (S−s−U+^var^ phenotype) and encoded by a hybrid glycophorin gene [29]. Of these, ∼23% are associated with a variant GPB that usually expresses the He antigen, albeit variably [35]–[37]. Nucleotide changes in or around *GYPB* exon 5 were suggested to be its molecular origin [29]. Notably, a higher prevalence of the S−s−U− phenotype is found in Africa (2–8%); among the pygmies (20%) [38]–[40], up to 37% of West Africans and ∼1% of African Americans [41]. Africans also have a higher prevalence of the Henshaw phenotype (S−s−U+^var^ or GP.He phenotype) [27]. The prevalence of GP.He in African Americans is 3% and up to 7% in people of African origin in Natal, Brazil [27]. The existence of these GPB variants in people of African origin has led to the speculation that these variants may have been selected as a result of the relative resistance that they confer against *P. falciparum* malaria. Recently, a new *GYPB-A-B* recombinant allele (Morobe allele) was found in a highly endemic area in Papua New Guinea [42], but its precise protective effect has not been yet characterized. In South and SE Asia significantly higher prevalence of antigens carried by GPA/GPB hybrids, such as the MUT, MINY, HIL, Hop, St^a^, Mur and Mi^a^ antigens, are present (0.68–15% vs. 0% in Caucasian), some of which also affect the expression of the GPB S antigen on RBC surface [27]. All these recombinant variants, largely described by blood group scholars, are consistent with the results of a genome wide survey for Copy Number Polymorphisms (CNPs) in the human genome developed using Comparative Genome Hybridization [43], which identified the *GYPB* locus as a CNP in African populations. A recent study have identified the *P. falciparum* ligand for GPB, which raised the possibility that mutations in the gene encoding Glycophorin B in malaria endemic areas could affect susceptibility to malaria through the inability of the ligand to bind to the varied receptor [44].

The Brazilian population exhibits extensive polymorphism in blood group antigens. Although many of these are well documented [45]–[47], no studies have been done to specifically relate the frequencies of defined polymorphic blood group antigens that function as receptors for *P. falciparum* and the ability of the parasite to invade them. An uncontrolled GPB phenotype-based study of four different ethnic groups in Colombia, suggested an association between the GPB S−s+ variant and a greater resistance to malaria (*P. vivax* and/or *P. falciparum*) in people of African origin [48]. A study by Beiguelman et al. [49] in a rural area of Rondônia was not able to substantiate the studies in Colombia [48] as they did not find any significant associations between GPB SS, Ss or ss phenotypes and *Plasmodium* infection status. However, preliminary studies in four other endemic regions of the Brazilian Amazon including Porto Velho of Rondônia also observed higher frequencies of the GPB S+s+ phenotype among *P. falciparum* malaria patients from Belém and Rio Branco, while higher frequencies of the GPB S−s+ phenotype were found in uninfected blood donors from Belém, Porto Velho, and Rio Branco [50]. The discrepancy between the two studies in Rondônia was attributed to potential differences of the populations studied.

These preliminary studies pointed to a potential association between GPB S+ carriers and their *P. falciparum* infection status, although the significance of these results was impacted by the lack of control for ethnicity, a potential confounding factor. Therefore, our present study in Porto Velho, Rondônia was aimed to further establish whether molecular variation in the *GYPB* gene, particularly the one that generates the GYPB*S/s alleles, influences host susceptibility to infection with *P. falciparum*, taking into account the possible confounding factor of ethnicity. In addition, we interpreted our results in the context of the coding nucleotide diversity and haplotype structure of *GYPB*, which might also influence the prevalence of *P. falciparum* infections in this population.

## Materials and Methods

### Ethics Statement

The research reported here was approved by the IRB of each of the collaborating institutions Hemocentro, UNICAMP, State of Sao Paulo; the Faculdade de Medicina, São José do Rio Preto, State of Sao Paulo; and the Universidade Federal do Rio de Janeiro. The collaborating institutions are registered with the OHRP (FWA00007713; FWA00000377; and FWA00003452, respectively) and their IRB are also registered. The overall protocol was also approved by the IRB from the New York Blood Center (Protocol 415-05). A written informed consent was obtained from all adults, as well as from the parents or legal guardians of minors who participated in the present study.

### Study population

Two groups of individuals have been recruited for this study over the period of 2006–2007 in Porto Velho, Rondônia: 1) *P. falciparum* infected individuals; and 2) uninfected individuals who were born and/or have lived in the same endemic region for over ten years, were exposed to infection but have not had malaria in the past or over the 2–3 year study period. All consenting individuals have been interviewed and information regarding their gender, age, date of birth, place of birth, mother's name and maiden surname, ethnic origin of their parent and their grandparents, length of residence in their present locality, history of exposure and/or number of malaria episodes in the last 10 years, and past treatment for malaria were recorded.

Recruitment of *P. falciparum* infected individuals (N = 83) was done in the local healthcare clinic, Centro de Pesquisa em Medicina Tropical (CEPEM), Porto Velho – Rondônia. The consented individuals were of ranging age (18–62) and a female/male ratio of 27/56 (Table 1). The blood samples of the consented infected individuals were analyzed for *P. falciparum* parasitemia using Giemsa stained thick blood smears. The density of parasitemia in the infected individuals was recorded and expressed as the number of asexual *P. falciparum* per microliter of blood assuming a leukocyte count of 8000/µl. All patients with any symptoms of malaria and/or microscopically confirmed infections were given standard and appropriate treatment. The *P. falciparum* treatments involved artemether-lumefantrine (Coartem®) or quinine-doxycycline therapy, the first-line anti-malarial therapies recommended by the Brazilian Ministry of Health, which are superior to other available and affordable treatments. DNA prepared from whole blood samples was later analyzed by PCR for parasite species specific genotyping using established protocols [51] to confirm their infection status with the malaria parasites. It appeared that only 32.3% of all the *P. falciparum* infected individuals had mono-infection with *P. falciparum*; the majority of the individuals had mixed infection with *P. vivax* (66.3%) or had a triple infection with *P. vivax* and *P. malariae* (1.4%).

**Table 1:** Demographic characteristics, ancestry estimations and *GYPB* * *S/s* genotype frequencies in cases and controls, tests for Hardy-Weinberg equilibrium and association between *GYPB* * *S/s* genotype frequency and infection with malaria.

	Mean Age (SD*)	Females/Males	African ancestry (SD)	European ancestry (SD)	Native American ancestry (SD)	SS (%)	Ss (%)	ss (%)	Significance of test for Hardy-Weinberg equilibrium
Controls (n = 199)	28.29 (9.16)	97/102	0.18 (0.14)	0.54 (0.19)	0.28 (0.17)	15 (7.54)	84 (42.21)	100 (50.25)	*P* = 0.65
Cases (n = 83)	31.76 (11.99)	27/56	0.18 (0.14)	0.54 (0.18)	0.28 (0.18)	3 (3.61)	55 (66.27)	25 (30.12)	***P*** **<0.01**
Total (n = 282)	29.30 (10.16)	124/158	0.18 (0.14)	0.54 (0.19)	0.28 (0.17)	Association test assuming dominance of S: ***P*** **<0.02** **			

*SD, standard deviation.

**Association persists (*P*<0.02) if age, gender and European, African or Native American ancestry are included as covariates.

Active recruitment of the uninfected individuals who were born and/or have lived in the same endemic region for over ten years and have reported that they never had an episode of malaria although their family members or neighbors had it (markers for exposure) was done in the villages around Porto Velho (Ouro Preto do Oeste, Guajará-Mirim, Ji-Paraná and Candeias do Jamari). Those who met the inclusion criteria and consented to participate were tested on site with the OptiMal® (DiaMed AG, Switzerland), a rapid malaria diagnosis test, to validate their non-infection status. Only those that were negative by the OptiMal® kit were bled. To further verify the non-infection status of the individuals in this study group, the DNA extracted from their whole blood sample was later analyzed by PCR for parasite species specific genotyping to identify those that might have asymptomatic malaria infections or to confirm their malaria infection free status using established protocols [51]. It appeared that 30 of the individuals were PCR positive for malaria DNA; 27 of them had *P. vivax* specific DNA and 7 had a mixed *P. vivax* and *P. falciparum* DNA. These individuals were therefore excluded from the uninfected control group but also were not included in the infected group. The final control uninfected group constituted of 199 individuals; 18–56 years of age and a female/male ratio of 97/102. A follow up of these individuals 2 years after the start of the study confirmed that they still had no episodes of malaria in the past or over the study period.

### Phenotyping for GPB Ss blood group antigens

The presence of GPB Ss antigens on the surface of the RBCs was detected by the hemagglutination test using specific gel cards (Diamed AG, Morat, Switzerland) and appropriate commercial antibodies. The testing was done using fresh blood from all individuals from both study groups at the Blood Bank of Porto Velho (Centro de Hemoterapia e Hematologia de Rondônia- Fundação Hemeron/Hemotereapy and Hematology Center of the Rondônia State – HEMERON Foundation) on the same day of the blood collection.

### Genotyping for GYPB*S/s

DNA samples of each individual were prepared from frozen blood samples. Genomic DNA was isolated by a whole blood DNA extraction kit (QIAmp, Qiagen, Valencia, CA) according to the manufacturer's instructions. The DNA solutions were analyzed for quality by agarose gel electrophoresis. The GYPB* S/s genotyping was performed using one or more of the following assays:

#### Allele-specific PCR

Allele-specific PCR (AS-PCR) for the GYPB*S/s alleles were performed in all samples. The sequences of primer combinations and control primers that amplified an unrelated gene (human growth hormone gene) were previously published [29]. AS-PCR was carried out under the following conditions: 1× PCR buffer, 1.5 mmol/L MgCl_2_, 0.2 mmol/L dNTP mix, 100 ng of sense and antisense primers, 100 ng of control primers, 2.5 U *Taq* DNA polymerase and ∼50 ng of genomic DNA per 50 µl of total volume. The amplification was performed using a 35-cycles protocol, with an annealing temperature of 62°C.

#### *GYPB* Exon 5 combination AS/PCR-RFLP assay

To determine if the S allele was silenced, genomic DNA samples from S−s+ samples genotyped as GYPB*S/s were amplified with the GPB4/5, GPBIVS5 and GPB5T primers [29], using a combination AS/PCR-RFLP assay to determine whether *GYPB* is present or absent and to distinguish the variant *GYPB* genes products in S−s+ (GYPB*S silent gene) individuals. The PCR products were digested with *Eco*RI overnight at 37°C. The uncut and digested products were analyzed on a 10% polyacrylamide gel.

#### BeadChip DNA analysis

For some of the *P. falciparum* infected and controls samples for which there was discordance between the two genotyping methods described above and the phenotyping by hemagglutination were re-tested for the GYPB*S/s genotypes using a DNA array, BeadChip™ Human Erythrocyte Antigen (“HEA”), containing specific probes directed to polymorphic sites in *RHCE*, *FY* (including *FY-GATA* and *FY265*), *DO* (including *HY and JO*), *CO*, *DI*, *SC*, *GYPA*, *GYPB* (including markers permitting the identification of U-negative and U-variant types), *LU*, *KEL*, *JK*, *LW* and one mutation associated with hemoglobinopathies (HgbS) (BioArray Solutions, Warren, NJ, USA). The HEA BeadChip assay was performed in accordance with a previously described protocol [52], [53].

#### *GYPB* exon sequencing of *GYPB*

Specific PCR amplification of *GYPB* is difficult because of its high homology with the two distinct glycophorin genes, *GYPA* and *GYPE*. We therefore designed the *GYPB*-specific primers using the following procedure: we aligned human *GYPA* (GenBank m60707), *GYPB* (GenBank m60708), and *GYPE* (GenBank m29609) sequences and identified sites that are variable and specific for *GYPB* gene segments encompassing exons 2, 4, 5 and 6. The *GYPB*-specific primers had to be at their 3′ terminals sites 100% specific for their own sequences. After PCR and sequencing, we verified the identity of the PCR amplicon by verifying the presence of *GYPB* specific sites. Primers used for PCR amplification contained a M13F or M13R tails (Table 2) and their PCR products included the respective exon and part of its flanking intron regions. PCR amplification was done using 10 ng of genomic DNA, 200 nM of each primer and Platinum PCR SuperMix (Invitrogen, Carlsbad, CA, US) in final volume of 50 µL. The cycling profile used was 2 minutes at 94°C, 45 cycles of 30 seconds at 94°C, 45 seconds at annealing temperature, 2 minutes at 72°C and a final extension of 5 minutes at 72°C. PCR products were purified and bi-directionally sequenced using M13 forward or M13 reverse primers and Applied Biosystem technology (Genewiz, NJ, US). Sequences were analyzed using phred-phrap-consed software [54] and the pipeline described by Machado et al. [55], using the Genbank sequence NC_000004.11 as reference.

**Table 2:** Primers designed to amplify *GYPB* exons 2, 4, 5 and 6 by PCR.

Specificity	Primer name	Sequences (in bold are the M13F or M13R tails of primers)	Annealing temperature	Size of PCR product
Exon 2	**M13F**-Exon2-for	**TGTAAAACGACGGCCAGT**GGACTGGAGGGATGTGAGA	55°C	402 bp
	**M13R**-Exon2-rev	**CAGGAAACAGCTATGACC**TAGAATTCCTCTGTAGTAA		
Exon 4	**M13F**-Exon4-for	**TGTAAAACGACGGCCAGT**GCATGGGACTGGCATCTC	60°C	722 bp
	**M13R**-Exon4-rev	**CAGGAAACAGCTATGACC**GCTTGGCCTCCCAAAATTATA		
Exon 5	**M13F**-Exon5-for	**TGTAAAACGACGGCCAGT**ATAGTATGTTAACTGTACTTTG	48°C	393 bp
	**M13R**-Exon5-rev	**CAGGAAACAGCTATGACC**TCTATGTGTCCAGTTGAAAA		
Exon 6	**M13F**-Exon6-for	**TGTAAAACGACGGCCAGT**CAGAGGCTGAAGTGGAGTCT	55°C	276 bp
	**M13R**-Exon6-rev	**CAGGAAACAGCTATGACC**TAGAGAATACAGTAATAGTG		

#### Genotyping a *GYPB* tagSNP using Taqman real time PCR assay

To complement the sequencing data and obtain a better coverage of the *GYPB* haplotype structure, we identified SNPs from the HapMap (June 2009) database that were not included in the re-sequenced regions (for example, intronic SNPs), were common (MAF>0.10) and were polymorphic in at least one of the HapMap population. We analyzed the pattern of linkage disequilibrium across *GYPB* from HapMap data and selected two tag-SNPs (that can be used as surrogate for untested SNPs, due to the pattern of linkage disequilibrium): rs4835511 and rs9685167. We genotyped them by TaqMan assays (Applied Biosystems, Palo Alto, CA, US); rs9685167 genotyping did not work properly, while rs4835511 did using 10 ng of genomic DNA, TaqMan® Genotyping Assay 20× and TaqMan® Genotyping Master Mix (Applied Biosystems®, Foster City, CA, US) in final volume of 10 µL.

### Ancestry Informative Markers genotyping

We genotyped 62 Ancestry Informative Markers (AIMs) in the DNA samples of all cases and controls. The first set of AIMs consisted of 14 SNPs reported and genotyped in two multiplex reactions as in Da Silva et al. [56] The second set of AIMs included 48 INDELs reported and genotyped in three multiplex reactions as in Santos et al. [57].

### Statistical and population genetics analyses

We used the Fisher exact test to assess the Hardy-Weinberg equilibrium. To measure association between S/s genotypes and malaria infection we used the haplotype score test by Schaid et al. [58] and Lake et al. [59] implemented in the software *Haplostats* v.1.4, assuming dominance for the S allele and when necessary, including age, gender, and African, European or Native American ancestry (see below) as covariates. Although *Haplostats* has been developed keeping in mind haplotype analyses, the association test (analogous to Fisher exact tests) is also suitable for single SNPs.

Individual European, African and Native American ancestry were estimated using the Bayesian clustering algorithms developed by Pritchard and implemented in the program STRUCTURE v2.3.2 [60], [61]. We assumed that three parental populations (K = 3 clusters) contributed to the genome of the admixed individuals. STRUCTURE estimates individual admixture conditioning on Hardy-Weinberg and linkage equilibrium on each of the K = 3 clusters, that represent the parental populations. We run the program using a length of burn-in Period of 100 000 and 10 0000 repetitions of MCMC after burning. We used prior population information for individuals from the parental populations to assist clustering (USEPOPINFO = 1) and assumed the admixture model for individuals from the admixed populations, inferring the alpha parameter for each population. We also used the parameters GENSBACK = 2 and MIGRPRIOR = 0.05. Moreover, we assumed that allele frequencies were correlated (i.e. similar across parental populations) and that the parental populations show different levels of differentiation (F_ST_, with prior mean of 0.01 and standard deviation of 0.05). The admixture in each group (cases and controls) is calculated by Structure as the average of the admixture for each individual within the group. We performed these analyses with the two sets of data we produced for this study: (1) the 48 INDELS previously used by Santos et al. [57], using the ancestral populations reported in that publication, and (2) using 62 AIMs (those in Santos et al. [57] and the 14 SNPs reported by da Silva et al. [56], using the parental populations from the latter publication. Both measurements of ancestry were highly correlated (Spearman correlation coefficients: 0.82, 0.89 and 0.92 for African, European and Native American admixture, respectively, with *P* always<0.01).

We inferred haplotypes considering SNPs with a Minor Allele Frequency (MAF) ≥0.05, using the method by Stephens and Scheet [62]. The recombination parameter ρ was also calculated for each population by using the method of Li and Stephens [63]. These inferences were performed by the software Phase v.2.1.1., using 10.000 iterations, thinning intervals of 100 and burn in of 1000. Linkage disequilibrium (LD) was estimated by r^2^ for SNPs with MAF≥0.05 in at least one population [64] and its significance assessed by LOD scores, using software Haploview v.3.2 [65].

For the analyses of the sequencing data, we assessed intra-population variability using the following estimators of the θ parameter based on the infinite-site-model of mutations: π, the per-site mean number of pair-wise differences between sequences [66], and by θ_w_, based on the number of segregating sites [67]. To investigate if the observed patterns of variability in the studied Brazilian population is consistent with the neutral model of evolution, we used the statistical tests of Tajima's D [68], Fu and Li's D* and Fu and Li 's F* [69] on the re-sequencing data, testing these statistics against the standard null hypothesis of neutrality (no natural selection) under constant population size.

## Results and Discussion

### GYPB*S/s frequencies in the control and *P. falciparum* infected populations

Two groups of individuals were studied; the uninfected group (N = 199) and the *P. falciparum* infected group (N =  83). Host DNA from these individuals was used for GYPB*S/s genotyping by AS-PCR, PCR-RFLP and/or DNA array analysis. Specific PCR amplification of GYPB*S/GYPB*s is difficult because of the high homology between *GYPA*, *GYPB* and *GYPE*. To have accurate results we have chosen two different methods for GYPB*S/GYPB*s genotyping: the allele-specific AS-PCR, an “in house” method, and the HEA (i.e. Human Erythrocyte Antigen) BeadChip, a microarray method commercially available. All samples were analyzed by both methods. When the genotype results in an individual were inconsistent using both methods, we excluded these individuals from further analyses, even thought the HEA BeadChip is known to be more accurate than the AS-PCR [52], [53]. Only 3 individuals had mismatching genotypes and were not included in the association studies. Genomic DNA samples from individuals phenotyped S−s+ but genotyped as GYPB*S/s were analyzed using a combination AS/PCR-RFLP assay [29], [70] in order to determine if the S allele was silenced (GYPB*S silent gene).

When the genotype distributions of GYPB*S/s alleles in the two study groups were compared, the differences in the frequencies of the GYPB*S/GYPB*S and GYPB*S/GYPB*s vs. GYPB*s/GYPB*s genotypes between the *P. falciparum* infected individuals (cases) and the uninfected individuals (control) were significant (69.87% vs. 49.75% of GYPB*S/GYPB*S and GYPB*S/GYPB*s and 30.1% vs. 50.25% of GYPB*s/GYPB*s, respectively; *P*<0.02, Odds Ratio = 1.55 with 95%CI = [1.02, 2.38]) (Table 1). In these analyses, we assumed that the presence of the GYPB*S allele is a dominant risk factor for susceptibility to infection (i.e., regardless if it is a homozygote or heterozygote), as it results in the phenotypic expression of GPB S+ on the surface of the RBCs. Intriguingly, only the infected individuals do not fit the Hardy-Weinberg expectation; with an excess of the heterozygous GYPB*S/GYPB*s genotype (66.27%, Table 1) as compared to what was expected (46.66%).

We observed a discordance of 5% between Ss phenotyping (performed in the field) and genotyping, which is compatible with previous studies [70]. Noteworthy, the results of phenotypes match those of genotypes, both for the excess of S+s+ or GYPB*S/GYPB*s heterozygous individuals among cases and for the association of the presence of the GPB S+ or GYPB*S putatively dominant allele with their infection status. Although *GYPB*, *GYPA* and *GYPE* genotyping is associated with technical difficulties due to their extensive sequence homology, the high concordance of our phenotype and genotype results and the significance of the association even when phenotypes are analyzed, suggest that our genotyping results are robust.

Interestingly, we have found a higher frequency of heterozygous GYPB*S/s genotypes among the *P. falciparum* infected individuals (66.27% in cases versus 42.21% in controls). Considering that heterozygous are more frequent that the homozygous S+s−, it seems that the amount of GPB S receptor molecules doesn't influence the susceptibility to *P. falciparum* infection. If we make an analogy to the Duffy blood group (FY), the receptor for *P. vivax*, and susceptibility of *P. vivax* malaria, different studies [20], [71], [72], including one in the Amazonian region of Brazil [73], have demonstrated that individuals with the *FYA/FYB* genotype have higher susceptibility to malaria infection. The *Fy* gene has two antigens (Fy^a^ and Fy^b^) that are encoded by the co-dominant alleles *FYA* and *FYB*, located on chromosome 1. The corresponding anti-Fy^a^ and anti-Fy^b^ antibodies define four different phenotypes; Fy(a+b+), Fy(a+b−), Fy(a−b+) and Fy(a−b−). The *FYA* and *FYB* alleles differ by one nucleotide change in exon 2 encoding glycine in Fy^a^ or aspartic acid in Fy^b^ at residue 42 [73]. In the Brazilian study [73], the authors reported a larger number of malaria episodes among patients with the heterozygote (FY*A/FY*B) genotype than the homozygote (FY*A/FY*A or FY*B/FY*B) genotypes. Individuals homozygous for *FYA* or *FYB* alleles expressed a lower quantity of the Duffy antigen, which is required for the *P. vivax* invasion, than those who were heterozygotes. Apart from the different levels of the expression, the specific conformation of the Fy^a^ and Fy^b^ antigens may also determine differences in the susceptibility to infection. Nevertheless, it was concluded that one of the possible consequences of differential susceptibility to *P. vivax* malaria could be modifications in allelic frequencies of FY*A and FY*B in populations exposed to *P. vivax*, the most prevalent malaria species in the Brazilian Amazon region.

Our study has verified for the first time that molecular variation in the *GYPB* gene, particularly in the GYPB*S/s alleles, influenced host susceptibility to infection with *P. falciparum* in Porto Velho, Rondônia. In this study we took into consideration the possible confounding factor of ethnicity by performing association analyses adjusted for admixture, as well as for age and gender (Table 1). An association between other human receptor polymorphisms and variations in the parasite ligands of *P. falciparum* that modulate susceptibility to malaria, was also demonstrated [25], [74]–[76].

**Figure 1:**
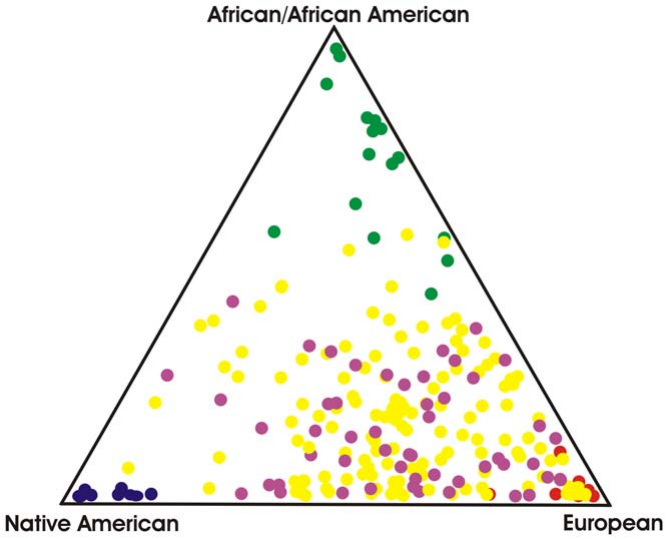
Estimation of admixture using Ancestry Informative Markers genotyping. Individual European, African and Native American ancestry were inferred from 60 ancestry informative markers in cases (magenta) and controls (yellow). Admixture was inferred by comparison with individuals from the putative parental populations: Europeans (red), African/African American (green) and Native Americans (blue). Admixture was estimated using the software Structure and average admixture over cases and controls is shown in Table 1.

GYPB*S/s allele frequencies vary across human populations; the GYPB*S allele (supposedly associated with infection) is less common in East Asians (∼10%) than in Sub-Saharan Africans (>25%) and Europeans (∼30–40%). Therefore, if the cases have more European ancestry than controls; a false positive result may emerge due to the association of any variant more common in Europeans, and thus the association results we got might not be at all related with susceptibility to infection conferred by the GPB Ss blood group antigens. To avoid a false positive result, we also measured the association among GPB Ss variants and infection by controlling the effect of admixture. Because we ascertained that African, European and Native American admixture do not differ among cases and controls (Figure 1 and Table 1), we exclude the possibility that our result is a spurious association. Controlling for admixture is essential in genetic epidemiology studies performed in Latin American populations, where large inter-individual differences in admixture are the rule [27].

We hypothesize that this Met29Thr polymorphism might be associated with changes in the structure of the GPB molecule that is used by *P. falciparum* to enter the RBCs. By having a Thr residue (GPB s+) on the RBCs instead of a Met residue (GPB S+), GPB s+ gains potentially a new site for O-glycosylation, which can likely alter its conformation and thus influence the efficiency of invasion by the parasites and ultimately, susceptibility to infection. Alternatively, this polymorphism may also affect dimerization of GPB molecules with other GPB or with GPA molecules [77].

### Population genetics of *GYPB* and inferences about natural selection

In principle, the GYPB*S/s alleles might not be the only functional GPB variants that modify *P. falciparum* invasion efficiencies in the Brazilian population studied. To understand the relationships between the observed association and the haplotype structure of *GYPB* in this population, we sequenced exons 2, 4, 5 and 6 of *GYPB* and their flanking regions, for a total of 1492 bp in sub-samples of cases and controls, matching the proportion of SS, Ss and ss genotypes observed in the total number of cases and controls studied. These sequences are publicly available under the GenBank accession numbers HQ639948–HQ640229. This sub-sample includes 41 cases (2 SS, 26 Ss, 13 ss) and 100 controls (8 SS, 42 Ss and 50 ss). We also used HapMap data available in June 2009 to explore the pattern of linkage disequilibrium across *GYPB* and selected the tag-SNP rs4835511 to be genotyped in the *GYPB* sequenced individuals using *TaqMan* (Applied Biosystem) assay.

**Figure 2:**
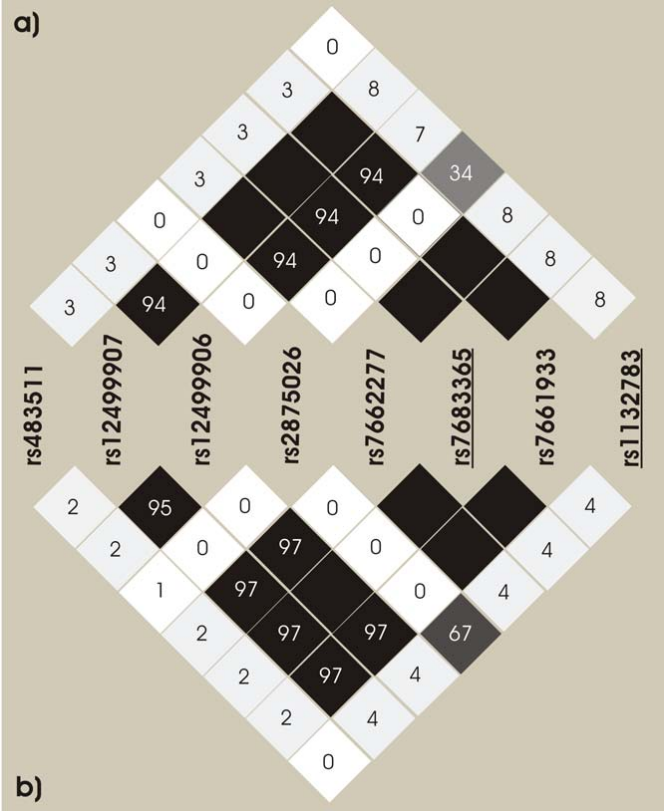
Linkage disequilibrium among common SNPs in *GYPB*. Linkage disequilibrium among common SNPs in *GYPB* was estimated in both study groups: the controls (a) and in the cases, malaria infected individuals from Brazilian Amazon (b). Underlined SNPs are non-synonymous substitutions: rs7683365 is the SNP determining S/s antigens; rs1132783 is a Ser/Thr polymorphism (see Table 3).

Table 3 shows the common *GYPB* haplotypes (i.e. a combination of alleles on the same chromosome) based on eight common SNPs across the sequenced region and the tag-SNP rs4835511. Haplotypes are sorted on the basis of their S/s (rs7683365) allele, and Figure 2 shows that the S/s SNP is in linkage disequilibrium with some common silent polymorphisms in exon 4 and its adjacent introns, but not with all the common SNPs reported in the *GYPB* sequenced regions. In particular, there is no linkage disequilibrium between the S/s alleles (rs7683365) and the non-synonymous common SNP rs1132783 (Ser/Thr) in exon 5. Since this polymorphism is predicted by PolyPhen [78] as benign, we assumed that its role in determining susceptibility to malaria infection is minor in respect to the S/s allele; SNP rs1132783 is located in the transmembrane domain of the protein, thus unlikely to have an effect on the host-parasite interaction. The site-specific PolyPhen algorithm (http://genetics.bwh.harvard.edu/pph/), which uses protein structure and/or sequence conservation information from each gene to predict whether a nonsynonymous mutation is “benign,” “possibly damaging,” or “probably damaging”, was shown to be the best predictor of the fitness effects of nonsynonymous mutations in a study analyzing a large polymorphism data set from 301 human genes [79].

**Table 3:** *GYPB* haplotype frequencies determined on the re-sequencing panel on the basis of common SNPs (MAF>0.05).

	rs4835511	rs12499907	rs12499906	rs41338748	rs7662277	rs7683365 a	rs7661933	rs1132783 b	Cases	Controls	Total
**Ancestral allele**	C	T	T	T	T	C	T	G			
**GYPB-s1**	T	.	.	.	.	.	.	.	5	13	18
**GYPB-s2**	.	.	.	.	.	.	.	.	37 c	109	146
**GYPB-s3**	.	.	.	.	.	.	.	C	4	0	4
**GYPB-s4**	.	.	.	A	.	.	.	C	6	19	25
**GYPB-s5**	.	.	.	A	.	.	.	.	0	1	1
**GYPB-S6**	.	G	.	.	A	T	A	.	1	1	2
**GYPB-S7**	.	.	G	.	A	T	A	.	0	1	1
**GYPB-S8**	.	G	G	.	A	T	A	.	26	49	75
**GYPB-S9**	.	G	G	A	A	T	A	.	3	7	10
**Number of chromosomes**									**82**	**200**	**282**

a SNP accounting for S (Thr) and s (Met) phenotypes.

b Ser(G)/Thr(C).

c The modal haplotype in each group is underlined.

Non-synonymous substitutions are underlined.

The pattern of nucleotide diversity of *GYPB* revealed by the sequencing data is also informative about the role of the S/s alleles in susceptibility to malaria infection, because it allows inferences about the action of natural selection driven by malaria during the human evolutionary history [80]. Inferences about the action of natural selection have two implications. First, variants on genes inferred to be under selection have contributed to determine phenotype variability and perhaps, differential susceptibility to diseases such as malaria. Second, by definition of natural selection, these variants have been associated with relatively different reproductive efficiencies (i.e. *fitness*) of their carriers, and therefore, they have biomedical relevance. In particular for malaria, Ayodo et al. [81] have evidenced that combining information from association studies and evolutionary inferences about natural selection increases the probability of identifying susceptibility genes of malaria infection.

Analysis of *GYPB* nucleotide diversity in the Brazilian population studied (Table 4) reveals that: (a) *GYPB* shows a level of diversity that is similar to the most variable loci (*ABO*, *SERPINA5*) observed in African populations [82]; the most diverse continental human population. This is due in part to the genomic structure of the glycophorins' region, that encodes the *GYPA*, *GYPB* and *GYPE* highly homologous genes, and which might be affected by high rates of gene conversion among these loci. The observed high diversity may also be due the admixed nature of the Brazilian population, whose diversity reflects the combining effect of the parental population's diversity. (b) When the diversity of *GYPB* is measured separately for haplotypes carrying S or s alleles, diversity is consistently lower across cases and controls for the s haplotypes (associated with resistance to infection) than for the S haplotypes (associated with infection), notwithstanding the higher frequency of the s allele, that being the most common in humans, is expected to be the ancestral one, a condition typically associated with higher nucleotide diversity.

**Table 4:** Summary of *GYPB* diversity indexes and tests of neutrality based on re-sequencing data of a subset of cases and controls and their partitions in S and s alleles (rs7683365) of the Ss blood group antigens.

Populations	Controls*	Controls-S	Controls-s	Cases*	Cases-S	Cases-s
Number of chromosomes	200	58	142	82	30	52
Segregating sites	26	17	8	21	15	4
Singletons	17	16	5	13	14	2
Non-synonymous (total/singletons)	5/3	3/3	1/0	5/3	3/3	1/0
ρ (per adjacent sites ×10^3^)	0.32	-	-	0.02	-	-
*θ* estimators						
π ± SD (×10^3^)	1.80±0.13	0.51±0.29	0.39±0.06	2.09±0.24	0.75±0.53	0.40±0.09
θW ± SD (×10^3^) (per site)	2.97±0.85	2.46±0.87	0.97±0.40	2.83±0.92	2.54±0.99	0.59±0.33
*Neutrality tests*						
Tajima's D	−1.092	**−2.413** b	−1.376	−0.777	**−2.372** b	−0.720
Fu and Li's D*	**−5.400** a	**−5.355** a	**−3.002** c	**−3.578** a	**−3.972** a	−1.217
Fu and Li's F*	**−4.385** a	**−5.138** a	**−2.891** c	**−3.035** a	**−4.071** a	−1.243

*The samples of cases and controls were selected so the proportion of SS, Ss and ss genotypes observed in the total set of cases and controls was matching.

a *P*<0.02,

b *P*<0.01,

c *P*<0.001.

The pattern of genetic diversity on a specific genomic region depends both on the demographic history of populations, as well as on locus specific evolutionary factors such as mutation, recombination and natural selection. Almost 60 years ago, Haldane [83] proposed the so-called “Malaria Hypothesis” - that malaria might act as a selective force on human populations. Since then, several studies have tested and verified this hypothesis in the human host [81] and the malaria parasites [84], [85]. To infer if malaria-driven natural selection has shaped the diversity of *GYPB*, we used statistical tests of the null hypothesis of neutrality: that the *GYPB* pattern of diversity may be explained considering only the demographic history of the studied population and mutation and recombination patterns of *GYPB*, without the need to invoke the action of other factors such as natural selection. These statistical tests [68], [69] (Tajima's D and Fu-Li's D* and F*) are based on the proportion of rare and common polymorphisms expected in a population under neutrality, and this proportion is informative about natural selection. Table 4 shows that Tajima's and Fu-Li's statistical tests are negative and significantly different from 0, which is indicative of an excess of rare alleles in respect to neutral expectations. This result is consistent with the following scenario involving the action of natural selection (i.e. a *selective sweep*): a beneficial substitution (putatively the s allele) rapidly increases in frequency (i.e. incomplete sweep) carrying its associated haplotype through a hitchhiking effect. This process, driven by natural selection, is too rapid for recombination to shuffle the surrounding haplotype. As a consequence, it is expected that the nucleotide diversity (π) of the haplotypes carrying the beneficial allele (s in this case) is lower than the alternative allele (S), as observed in our data (Table 4). During a selective sweep, rare substitutions become common both because: the rise in frequency of the s haplotype; other substitutions associated with S become rare; and new (and therefore rare) substitutions appear in the expanding positively selected haplotype. The negative values observed for the neutrality tests for *GYPB* is consistent with this scenario (Table 4).

In the case of the Brazilian admixed population, the observed excess of rare alleles in *GYPB* can not be a consequence of admixture, which generally results in a reduced proportion of rare alleles in respect to neutral expectations (and therefore, to positive values of statistical tests such as D, D* and F*) [69]. Instead, we are likely observing the signature of a selective sweep that occurred during the last thousands of years of the human evolution in malaria affected regions mainly in Southern Europe and more likely Africa [86], where the ancestors of our studied Brazilian population settled (see Table 1 for the predominant European admixture proportion). Although our interpretation may be true and consistent with the results of the present association study, an unambiguous inference about the action of natural selection would require a comparison of *GYPB* diversity with a set of other loci not affected by natural selection, that would allow to obtain a more realistic null neutral distribution of neutrality statistics (D, D*, F*) that incorporate the specific demographic history of the studied population. On the other hand, the lack of evidence of the action of natural selection on African or European populations on genomic screenings of signatures of natural selection [87]–[89] may be due to the difficulties in genotyping/sequencing of the glycophorins' genomic regions.

Altogether, our results suggest that the S domain on GPB is important for its binding to the specific ligand of the *P. falciparum* parasite, EBL-1, which was recently identified and characterized [44]. RBCs carrying glycophorin B but not RBCs lacking glycophorin B (S−s−U−) were shown to adsorb the native EBL-1 from *P. falciparum* culture supernatants. Future studies are needed to demonstrate whether EBL-1 binds differentially to GPB S−s+ vs. S+s+ RBCs, and thus indirectly substantiate at a molecular level our observation of association studies at the population level and our population genetics inferences about the action of natural selection. Performing similar studies in other regions of the Amazons and other endemic regions of the world is also needed to further substantiate our observations; including both association studies with larger sample sizes and with a population genetics approach that include sequencing and genotyping at a higher resolution. Because *P. falciparum* shows also high population structure, in particular in the Amazon Region [90], it is also important to understand the extent of variability in host-parasite interaction and their co-evolution. The statistical association between the presence of the S allele and infection supports also the hypothesis that *P. falciparum* parasites in the Brazilian Amazon regions utilize GPB as a key receptor for invasion, and consequently individuals who carry distinct *GYPB* gene variants, which might facilitate erythrocyte invasion, will be more susceptible to *P. falciparum* infection. Thus, our results reinforce the need of studies focusing on *in vitro* invasion assays using erythrocytes with diverse *GYPB* genotypes and *P. falciparum* strains from different origin to establish the role of the GPB receptor for *P. falciparum* parasites of this region.
